# Radical “Visual Capture” Observed in a Patient with Severe Visual Agnosia

**DOI:** 10.1155/2003/404087

**Published:** 2003-01-01

**Authors:** Akiko Takaiwa, Hirokazu Yoshimura, Hirofumi Abe, Satoshi Terai

**Affiliations:** ^1^Department of RehabilitationHakujuji HospitalJapan; ^2^Department of PsychologyMeisei UniversityJapan; ^3^Department of RehabilitationFukuoka Tokushukai HospitalJapan; ^4^Department of NeurologyHakujuji HospitalJapan

**Keywords:** visual capture, visual agnosia, left posterior cerebral occlusion, cross-modal integration

## Abstract

We report the case of a 79-year-old female with visual agnosia due to brain infarction in the left posterior cerebral artery. She could recognize objects used in daily life rather well by touch (the number of objects correctly identified was 16 out of 20 presented objects), but she could not recognize them as well by vision (6 out of 20). In this case, it was expected that she would recognize them well when permitted to use touch and vision simultaneously. Our patient, however, performed poorly, producing 5 correct answers out of 20 in the Vision-and-Touch condition. It would be natural to think that visual capture functions when vision and touch provide contradictory information on concrete positions and shapes. However, in the present case, it functioned in spite of the visual deficit in recognizing objects. This should be called radical visual capture. By presenting detailed descriptions of her symptoms and neuropsychological and neuroradiological data, we clarify the characteristics of this type of capture.

## 1. Introduction

Using a lens that reduces the size of an object's retinal image we can present an observer with contradictory visual and tactual information. If he grasps an object while viewing it through the reducing lens, vision should tell him the object is of a certain size and touch should tell him it is much larger. In this situation we usually perceive the visually observed size as real, which is called visual capture and is demonstrated experimentally by Rock and colleagues [10,11]. Generally speaking, visual capture is the phenomenon that when vision and touch provide discrepant information about the position, shape, or size of an object, our perception of the object is dominated by visual information.

It may be reasonable to think that visual capture functions when vision presents some clear percept contradicting the tactual one. We, however, encountered a patient who showed a peculiar visual capture. She suffered from a cerebral infarction in the left occipital lobe and had difficulty in identifying common objects by vision but could easily identify them by touch. If given the task of identifying an object by vision and touch simultaneously, can she identify it as easily as in the case of the Touch-Only condition?

Several researchers have reported cases in which patients showed difficulty in identifying common objects by vision but did not show much difficulty in identifying them by touch. However, only a small number of researchers have examined the case in which patients use both vision and touch simultaneously. Table 1 shows the results of the studies by Ettlinger and Wyke [3] and DeRenzi et al. [2] in which they examined three conditions, namely, Vision-Only, Touch-Only, and Vision-and-Touch conditions. The scores of their patients under the Vision-and-Touch condition were between those

ISSN 0953-4180/03/$8.00 © 2003 – IOS Press. All rights reserved

Table 1
Scores of patients with visual agnosia in object-naming task reported previously

| Researchers | Age/Sex | Clinical diagnosis | Location of lesion | Vision | Touch | Vision+Touch |
|---|---|---|---|---|---|---|
| Ettlinger and Wyke(1961) | 59/M | Brain infarction | Left Occipital lobe(?) | 16/42 | 33/42 | 26/42 |
| | 65/M | Brain infarction | Left Occipital lobe | 8/60 | 45/60 | 38/60 |
| DeRenzi et al. (1987) | 66/M | Brain infarction | Left Occipital lobe | 31/60 | 43/60 | 40/60 |
| | 76/M | Brain infarction | Left Occipital lobe | 17/60 | 45/60 | 35/60 |

obtained under the Vision-Only and Touch-Only conditions. This implies the possibility that visual information somehow interferes with tactual information. In the context of visual capture, the problem can be formulated as follows: Does visual capture function even when vision itself cannot perceive concrete object?

Recently, intersensory relationships have been investigated not only from the psychological point of view but also from the neurophysiological or neuropsychological point of view. For example, Farnè et al. [4] demonstrated cross-modal extinction; the term 'extinction' refers to a clinical sign whereby the patient is able to detect a single stimulus presented to either the ipsi- or contralesional affected side of the body but fails to detect the stimulus when it is delivered to the contralesional affected side with a concurrent stimulus presented on the ipsilesional affected side. Rossetti et al. [12] proposed the idea of vectorial coding for the interpretation of data obtained from their prismatic visual and proprioceptive shift experiment. While through vision the subjects perceived some clear object in their experiments, our patient failed to perceive any of the concrete objects. The present research investigated the possibility of visual capture by imposing the Vision-and-Touch condition as well as the two single-modality conditions on our patient.

## 2. Methods

### 2.1. Patient

The patient was a 79-year-old right-handed female. She was an instructor of tea ceremony and had completed elementary education. She had been treated for hypertension and diabetes mellitus for about ten years. She had a sudden onset of headache at 3 pm on July 27, 1995, and the following day, she was observed dragging her right foot when walking. On July 29, she repeatedly talked about past episodes and told incoherent stories. She was admitted to the nearest hospital on the same day because of alteration of her state of consciousness and mild right hemiparesis, and was subsequently treated conservatively. Thereafter, she was referred to the first author's hospital and was admitted on September 25, 1995. Examination disclosed a blood pressure of 120/64 mmHg, and a regular pulse of 78 beats/min. The patient was alert with normal orientation. However, right homonymous hemianopsia, bilateral presbyacusis, mild right hemiplegia, right-sided mild hemiparesis, and right hemisensory impairment were noted. The right deep tendon reflex was enhanced and there were no pathological reflexes.

### 2.2. Tests and examinations

An intelligence test was administered using the Wechsler Adult Intelligence Scale Revised (WAIS-R) and the patient's language abilities were examined mainly using Western Aphasia Battery (WAB). In order to assess her fundamental visual abilities, the following examinations were carried out; sight and visual field examination, delineation of figures and objects, picture matching, visual discrimination, face recognition, and color perception.

The nature of visual capture was investigated by comparing her performance in object-naming tasks under the following three conditions: Vision-Only, Touch-Only, and Vision-and-Touch. Under each condition, the same 20 common objects were presented one by one and the patient was instructed to identify the object verbally. Each trial ended when the patient gave up and refused to continue the task. The three conditions were each tested on a different day to exclude carry-over effects. The order in which they were presented was Vision-and-Touch, Touch-Only, and Vision-Only. The Touch-Only condition was carried out using an opaque sack in which the target object was placed.

We investigated her neuroradiological state by magnetic resonance imaging (MRI) and Xe-enhanced computerized tomography (Xe-CT).

## 3. Results

### 3.1. Neuropsychological observations

Intellectual abilities were assessed by WAIS-R. Because her age was above the upper limit of the test, her scores were estimated based on the norm for the oldest age. The results showed that her verbal IQ was 81, which did not indicate apparent inferiority. Her performance, however, could not be assessed accurately because of visual agnosia. She was courteous throughout all the tests and had a complete comprehension faculty. Her memory span for digits was six forward and four backward.

Language abilities were tested mainly by WAB. While fluent in spontaneous speech, she had difficulties in finding the right words, and she could form only a few words in one minute for the word fluency task. In the speech repetition task, she could repeat sentences correctly. In the auditory comprehension task, the number of correct answers was 16 out of 20 yes/no questions. In addition, she was able to correctly identify the parts of her body and distinguish left from right relatively well. For the visually presented stimuli, however, her performance was severely impaired. When asked to select the correct picture of a common object from several candidates, she correctly responded to only one out of ten trials. Although she could follow the verbal instructions such as "Raise your hands" or "Close your eyes" generally, she did not respond correctly to the tasks necessary to access visual stimuli such as "Point to the pencil and the book".

In the reading task, when presented with a number or a word written in Chinese characters (kanji) or Japanese syllabary (hiragana) and instructed to read it aloud, she had extreme difficulty. However, when the *Schreibendes Lesen* technique was used, that is, when the stimulus syllabary was tactually presented on the patient's palm or the patient was permitted to trace the syllabary with her finger, her performance improved to some extent.

In the writing task, when shown an image of an object and asked to write the object's name, she could not do it at all. When words or sentences were presented orally, however, she could write them sometimes with mirror-like letters or in letter-by-letter manner. Although she was frequently aware that she was making errors, she could not correct them. When shown a syllabary and asked to copy it, she could not do the task.

She had trouble using common objects and dressing herself, for example, she mistook a pair of pants for a coat. These symptoms, however, should not be regarded as those of ideational apraxia or dressing apraxia, but should be regarded as results of the impairment of visual recognition itself. In the apraxia examination, she correctly performed the ideomotor apraxia test. She was able to use common objects correctly, when informed of their names or when given an appropriate auditory cue.

### 3.2. Fundamental visual abilities a) *Visual acuity and visual field:* Visual acuity was 0.01 for the right eye and 0.1 for the left eye, and she had right homonymous hemianopsia with macular sparing.
b) *Delineation of figures and objects:* Although she could replicate drawings by tracing, she failed to do so without tracing and her drawings from memory were very inaccurate.
c) *Visual matching of pictures:* When instructed to match the identical pictures among ten pictures of animals, she performed the task successfully (10/10). Visual discrimination: For two visually presented pictures, she could discriminate correctly nine items out of 10 trials. She could discriminate the size and distance of objects presented on the pictorial materials, and when walking, she could avoid obstacles standing in her path.
d) *Face recognition:* She had great difficulty recognizing her family's faces and distinguishing a male from a female. She also failed to recognize any photos of faces of well-known staff members.
e) *Color perception:* Although she could correctly discriminate three colors (black, yellow, and green) out of nine colors, she could not name any of them. When verbally instructed to group several common objects according to their color, however, she could do it fairly well (for example, "What color is the mailbox?") (7 correct answers out of 8). She could neither select nor paint the correct color of objects on the drawings even after she was given information about the objects.

### 3.3. Object naming tasks

We compared her abilities of object naming under the following three conditions: Vision-Only, Touch-Only, and Vision-and-Touch.

a) *Vision-Only condition:* Our patient identified correctly only 6 items out of 20 when they were presented only visually. She showed perseveration in wrong responses.
b) *Touch-Only condition:* This condition was applied by using a sack in which the target object was placed. By touching the object in the sack with her nonparalyzed left hand, she identified fairly well. That is, she could name 16 objects out of 20 correctly, with the tendency to name small

Table 2
Scores of the present patient in the object-naming task under three conditions

| Item | Vision | Touch | Vision+Touch |
|---|---|---|---|
| pencil | +(6) | +(2) | +(4) |
| ball | − | +(2) | − |
| newspaper | − | − | − |
| cup (glass) | +(5) | +(4) | − |
| yarn | +(4) | +(3) | − |
| stamp | − | − | − |
| eraser | − | +(2) | +(2) |
| comb | − | +(3) | − |
| pistol | − | +(83) | − |
| key | +(15) | +(5) | − |
| toothbrush | − | − | − |
| rubber band | − | − | − |
| hammer | +(1) | +(2) | +(5) |
| knife | − | +(2) | − |
| spoon | − | +(5) | +(3) |
| ashtray | − | +(43) | − |
| fork | − | +(30) | − |
| nail clipper | +(2) | +(37) | − |
| magnifying glass | − | +(35) | +(5) |
| clothespin | − | +(20) | − |
| correct rate | 6/20 | 16/20 | 5/20 |

+ represents correct response and − represents incorrect response. Numbers in parentheses represent the response time in seconds for correct responses.

objects incorrectly. When instructed to take out the target object from among five objects in the sack, she did so correctly for 17 out of 20 trials.

c) *Vision-and-Touch condition:* When presented an object and permitted to use vision and touch simultaneously, she could name correctly only 5 items out of 20, which was similar to the score under the Vision-Only condition. For some items, she had the chance to use an auditory cue to identify the objects. For example, when manipulating the pistol, she happened to pull the trigger. On hearing the click sound, she could identify it correctly.

The results showed significant differences in correct response rates between Touch-Only (16/20) and Vision-Only (6/20) ($\chi^2(1) = 8.19,\ p < 0.005$) and between Touch-Only (16/20) and Vision-and-Touch (5/20) ($\chi^2(1) = 9.63,\ p < 0.005$) and no significant difference between Vision-Only (6/20) and Vision-and-Touch (5/20) ($\chi^2(1) = 0.13,\ p > 0.50$).

The patient's responses in the naming of individual items under each condition are shown in Table 2. The total scores for each of the three conditions showed that the objects were well recognized by touch when presented without vision, but the performance was severely impaired and comparable to the performance under the Vision-Only condition when accompanied by vision. The individual responses, however, were not always consistent with this principle. She identified two items correctly under all three conditions, and she could not name four of the items correctly under any of the conditions. Except for these six items, considering the phenomenon of visual capture, the responses were expected to be as follows: Vision-Only condition, incorrect; Touch-Only condition, correct; and Vision-and-Touch condition, incorrect. Of the remaining 14 items, seven items fitted this expectation but the other seven did not, which suggests that the patient struggled to recall the objects' names. The following verbal responses indicate her state: For the item Key, she answered "Key" under the Touch-Only condition; "It may be a hammer. No. It's neither a nail clipper. Well, maybe a key" in the Vision-Only condition; "This came up last but I forget easily. How do you use this, is this just a moneybox, or something like ... Are you supposed to hang it? Hold it yourself, you hold it ..., what is it? You are, this being here means ..., then if you changed it like this, like you know, it becomes a problem. What is this? A kind of ... It has many uses. If you ask, this is a ... " in the Vision-and-Touch condition. Embarrassed by her performance, she showed an inconsistent performance, but the total scores and the results for half of the items (7/14) supported our definition of visual capture in these object-naming tasks.

### 3.4. Neuroradiological findings

MRI demonstrated mixed intensities on T2-weighted images in the left temporal and occipital areas, which are supplied by the left posterior cerebral artery (Fig. 1). A part of the occipital lesion was shown as hyperintense on a T1-weighted image. These findings indicate hemorrhagic infarction. A MR angiogram revealed severe stenosis of the interpeduncular segment of the left posterior cerebral artery without any other significant lesions, suggesting the possibility of recanalization of a once-occluded lesion. Xe-CT revealed the decrease in cerebral blood flow in both hemispheres. In particular, severely reduced cerebral blood flow was observed in the left temporal and occipital areas (Fig. 2).

## 4. Discussion

Visual agnosia is defined as the inability to recognize common objects which cannot be attributed to impaired visual function or language. It is defined further as cases in which the etiology of the impaired vi-

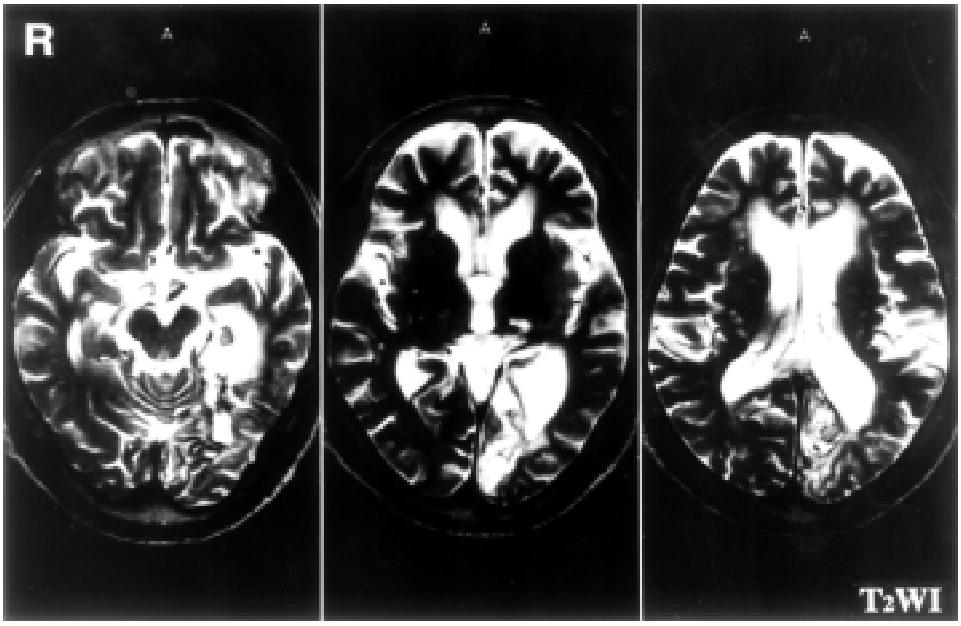

Fig. 1. T2-weighted MRI of the brain.

sual recognition of common objects is not attributed to aphasia, low intellectual capacity or disturbed consciousness, and the patient can recognize the objects via other perceptual processes.

The assessment of visual agnosia tends to center on the examination of the difference in the abilities of visual and tactual perceptions. Many authors have mentioned that the diagnosis can be made when the tactual perception is well preserved in spite of severely impaired visual perception. However, Morin et al. [8] have described cases of visual agnosia associated with tactile agnosia. As reported by them, their cases with impaired tactual perception in addition to visual agnosia might possibly be similar to those described in previous reports on visual agnosia. Neurological disorders, such as simultaneous impairment in both visual and tactual perceptions, had been designated as multimodal agnosia by Feinberg et al. [5].

On the other hand, the ability of our patient to recognize common objects by touch was rather good, but was markedly disturbed by vision. We first examined the difference in her visual and tactual object perceptions, and we found an apparent dissociation of correct responses, supporting the definition of visual agnosia. Our patient could recognize 16 out of 20 objects under the blindfold condition, while no more than five correct answers were obtained under the simultaneous stimuli of both visual and tactual perception. The disturbed tactual recognition under the simultaneous visual and tactual perceptions exerted an influence on the use of the object and made it impossible to manipulate properly. Although the abnormal behavior resembled the clinical symptom of apraxia, such as ideational and dressing apraxia, this possibility was ultimately discarded because the interception of vision made it possible to use the objects. In addition, she was able to use the objects correctly, when informed of their names or when given an appropriate auditory cue.

It seems difficult to make a diagnosis of visual agnosia in patients with impaired language function. The present case had transcortical sensory aphasia diagnosed based on results of the WAB test. In addition, in the finger-pointing task in the auditory comprehension test, our patient could hardly give the correct answer because the task was performed using visually presented stimuli. On the other hand, the number of correct responses increased in the yes/no questions (16/20), body-part identification, finger identification, and left-right discrimination tasks on WAB examination. The profile of the WAB test showed the decreased number of correct responses in the finger-pointing task that required vision. Kertesz et al. [7] described that 5 out of 15 cases with transcortical sensory aphasia were associated with visual agnosia, which indicates our patient is not a rare case.

Optic aphasia is known as one of the clinical entities causing impairments in identifying visually presented objects. Fundamentally, patients with optic aphasia

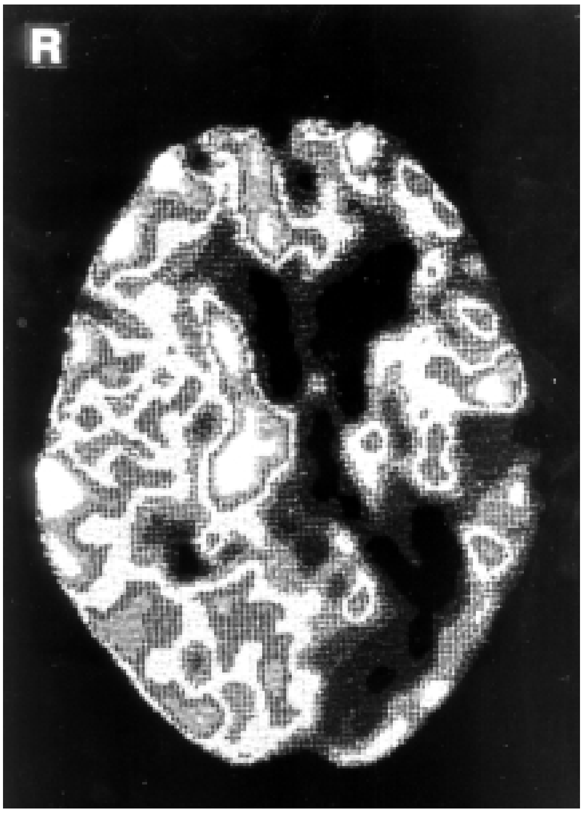

Fig. 2. Evaluation of cerebral blood flow using Xe-CT.

have preserved ability of object recognition. This symptom is characterized by the possibility of communicating the name of visually presented objects to the examiner by gestures or categorical classification, but it differs in our case.

Ettlinger and Wyke [3] and DeRenzi et al. [2] assessed the difference in object recognition abilities under various perceptual conditions; visual alone, tactual sensation alone, and the simultaneous stimuli of both vision and tactual sensations. In the case described by Ettlinger and Wyke [3], the task of naming objects was performed and the error rate was low in the order of tactual sensation alone, vision-and-tactual sensations and vision alone. DeRenzi et al. [2] reported 16 cases of occlusion of the left posterior cerebral artery, among which three had visual agnosia. The correct response in the task of naming objects tended to be more impaired under the condition of both vision and tactual sensations than under that of tactual sensation alone in the above three patients with visual agnosia.

In recent brain imaging studies, James et al. [6] showed that haptic exploration of novel three-dimensional objects produced activation, not only in the somatosensory cortex, but also in areas of the occipital cortex associated with visual processing. Calvert et al. [1] provided evidence of audiovisual crossmodal binding by convergence onto multisensory neurons in the human heteromodal cortex, which suggests that response enhancement and depression are a general property of multisensory integration operating at different levels of the neuroaxis and irrespective of the purpose for which sensory inputs are combined. Pavani et al. [9] demonstrated that sound localization performance of right-brain-damaged (RBD) patients with visuospatial neglect was severely impaired compared with that of RBD controls, particularly when sounds originated from the contralesional hemispace. These data suggest a closely related neural network between the visual and other modal loci in the brain.

In general, it may be considered that lots of perceptual information from visual, tactual and auditory sensations is integrated to represent a unified object. The mechanism, however, of how the information is integrated and controlled is not well known. Based on the above two reports of Ettlinger and Wyke [3] and DeRenzi et al. [2] and the present case, it may be presumed that there is more than one type of pathway for the tactual object recognition. One is that in which the visual information interferes with the tactual one, and the other is that without such interference. The present research showed a clear phenomenon that visual capture functions even when vision itself cannot perceive concrete object. Further investigations are necessary to clarify the brain mechanism of the capture and to explain why it is observed only between vision and touch, not between vision and audition.

## References

[1] G.A. Calvert, R. Campbell and M.J. Brammer, Evidence from functional magnetic resonance imaging of crossmodal binding in the human heteromodal cortex, *Current Biology* **10** (2000), 649–657.

[2] E. DeRenzi, A. Zambolin and G. Crisi, The pattern of neuropsychological impairment associated with left posterior cerebral artery infarcts, *Brain* **110** (1987), 1099–1116.

[3] G. Ettlinger and M. Wyke, Defects in identifying objects visually in a patient with cerebrovascular disease, *Journal of Neurology, Neurosurgery, and Psychiatry* **24** (1961), 254–259.

[4] A. Farnè, F. Pavani, F. Meneghello and E. Làdavas, Left tactile extinction following visual stimulation of a rubber hand, *Brain* **123** (2000), 2350–2360.

[5] T.E. Feinberg, L.J. Gonzalez-Rothi and K.M. Heilman, Multimodal agnosia after unilateral left hemisphere lesion, *Neurology* **36** (1986), 864–867.

[6] T.W. James, G.K. Humphrey, J.S. Gati, P. Sevos, R.S. Menon and M.A. Goodale, Haptic study of three-dimensional objects activates extrastriate visual areas, *Neuropsychologia* **40** (2002), 1706–1714.

[7] A. Kertesz, A. Sheppard and R. MacKenzie, Localization in transcortical sensory aphasia, *Archives of Neurology* **39** (1982), 475–478.

[8] P. Morin, Y. Rivrain, F. Eustache, J. Lambert and P. Courtheoux, Visual and tactile agnosia, *Revue Neurologique* **140** (1984), 271–277.

[9] F. Pavani, F. Meneghello and E. Làdavas, Deficit of auditory space perception in patients with visuospatial neglect, *Neuropsychologia* **39** (2001), 1401–1409.

[10] I. Rock and C.S. Harris, Vision and touch, *Scientific American* **216** (1967), 96–104.

[11] I. Rock and J. Victor, Vision and touch: An experimentally created conflict between the two senses, *Science* **143** (1964), 594–596.

[12] Y. Rossetti, M. Desmurget and C. Prablanc, Vectorial coding of movement: Vision, proprioception, or both? *Journal of Neurophysiology* **74** (1995), 457–463.
